# The Electro-Optical Performance of Silver Nanowire Networks

**DOI:** 10.1038/s41598-019-47777-2

**Published:** 2019-08-09

**Authors:** Hugh G. Manning, Claudia Gomes da Rocha, Colin O’ Callaghan, Mauro S. Ferreira, John J. Boland

**Affiliations:** 1 School of Chemistry, Trinity College Dublin, Dublin 2, Ireland; 2 Department of Physics and Astronomy, University of Calgary, 2500 University Drive NW Calgary, Alberta, T2N 1N4 Canada; 3 School of Physics, Trinity College Dublin, Dublin 2, Ireland; 4 Centre for Research on Adaptive Nanostructures and Nanodevices (CRANN) & Advanced Materials and Bioengineering Research (AMBER) Centre, Trinity College Dublin, Dublin 2, Ireland

**Keywords:** Nanowires, Computational methods, Electronic devices

## Abstract

Networks of metallic nanowires have the potential to meet the needs of next-generation device technologies that require flexible transparent conductors. At present, there does not exist a first principles model capable of predicting the electro-optical performance of a nanowire network. Here we combine an electrical model derived from fundamental material properties and electrical equations with an optical model based on Mie theory scattering of light by small particles. This approach enables the generation of analogues for any nanowire network and then accurately predicts, without the use of fitting factors, the optical transmittance and sheet resistance of the transparent electrode. Predictions are validated using experimental data from the literature of networks comprised of a wide range of aspect ratios (nanowire length/diameter). The separation of the contributions of the material resistance and the junction resistance allows the effectiveness of post-deposition processing methods to be evaluated and provides a benchmark for the minimum attainable sheet resistance. The predictive power of this model enables a material-by-design approach, whereby suitable systems can be prescribed for targeted technology applications.

## Introduction

**Figure 1:**
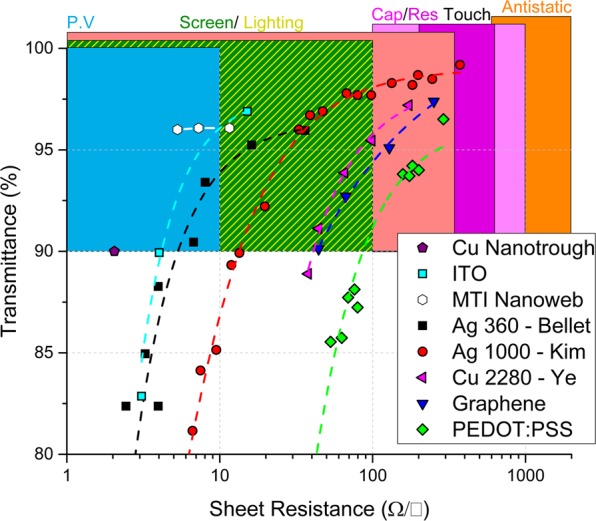
Sheet resistance (*R*_s_) requirements for various flexible transparent conductor (TC) applications, photovoltaic (PV), screen and lighting, capacitive and resistive touch screens and antistatic coatings (top). Transmittance, *T-R*_s_ graph, groups of data points compare the best performing (TC) technologies currently available. Cu Nanotrough & ITO^56^, MTI Nanoweb^57^, Ag nanowires (NWs) with an aspect ratio (AR) of 360^13^, 1000^58^, Cu NWs with an AR of 2280^9^, PEDOT:PSS TC^2^, calculated and experimental graphene TCs^58,59^.

Modern photovoltaics, light-emitting devices, touch screens and thin-film transparent heaters all rely on a transparent conductor (TC) layer for operation. The most commonly used material for TCs has been ITO (tin-doped indium oxide), however, the brittle nature of the ITO film makes it incompatible with flexible device platforms^1^. Moreover, the scarcity of indium and the high cost of the ITO film deposition has motivated the search for alternative materials, which now includes conductive polymers^2^, carbon nanotubes^3^, graphene^4^, metal mesh^5^, crackle networks^6^ and networks composed of metallic nanowires such as Ag, Au and Cu^7–9^. Nanowire networks (NWNs) have demonstrated excellent optical, electrical and mechanical performances through low-temperature high-throughput fabrication techniques such as spray deposition^10^, Mayer rod coating^11^, and roll-to-roll slot die printing^12^. In particular, Ag NWNs can not only match the electro-optical performance of ITO, but can also fulfil the demands for the emerging flexible electronics market^13^. Next-generation devices such as flexible solar cells^14^, touch screens^15^, displays^16^, thin-film heaters^17^, wearables^18,19^ and anti-static coatings^20^ require flexible electro-optical components. Each TC film requires a high optical transmittance value (*T* > 90%) whereas the electrical requirements of the sheet resistance (*R*_s_) is application-specific^4^. Figure 1 compares the current performance requirements of several proposed TC materials to ITO on a *T*-*R*_s_ curve against the back drop of the requirements for a range of technologies. It is clear that Ag NWNs can fulfil the required optical and electrical performances for many technologies by tuning the *R*_s_ value. In the case of ITO, the optical and electrical properties are modulated by changing the film thickness^21^. For Ag NWNs, performance depends on the wire density (*n*_w_), which, for the idealised case of a network made with wires of the same length and diameter is related to the junction density (*n*_j_), the nanowire length (*L*) and a contact probability (*P* = 0.2027) through Eq. 1 below^22^

1 $${n}_{w}=\sqrt{\frac{2{n}_{j}}{P\pi {L}^{2}}}$$

The network *R*_s_ is ultimately controlled by the junction resistance (*R*_jxn_) associated with overlapping Ag nanowires (NWs) in the network. *R*_jxn_ is a consequence of an electrically insulating few nm thick polyvinolpyrollidone (PVP) layer that forms a metal-insulator-metal configuration where ever NWs overlap to form a junction^23^. Modification of the PVP surface layer can give resistive switching memory effects^24,25^, or enhance the thermal and chemical stability of the Ag NWNs^17,26,27^.

PVP is necessary during the synthesis process and stabilises the nanowires in solution^28^. Optimisation of the *R*_jxn_ value in NWNs typically involves post-processing techniques such as thermal treatment^29^, mechanical pressing^30^, cold welding^31^, optically induced welding^32^, electrochemical Ostwald ripening^33^ or electrical stressing^34^, each of which can dramatically lower the *R*_s_. However, in the absence of an electro-optical predictive tool, the effectiveness of these processing methods has been difficult to assess, compare and prescribe for specific applications.

We previously introduced a computational approach to describe the  conduction properties of metallic NWNs using a multi-nodal representation (MNR) model which calculates the *R*_s_ considering the contributions associated with NW junctions (*R*_jxn_) and the NW segments (with inner resistances given by *R*_in_) between them^23^. One of the computational implementations for this model is available in the Supporting Information. Incorporating the inner-wire resistance (which depends on the NW material and diameter) is important but often overlooked. It allows the skeletal resistance of the network (in the limit when *R*_jxn_ → 0) to be determined, for which the resulting network is comprised of ballistic NW junctions, representing the ultimate conductivity of the NWN^23^. It is well known that increasing the NW length to diameter aspect ratio (AR) of the NWs results in lower *R*_s_ values and a larger *T*^35,36^. Thus far, electro-optical models of Ag NWNs have used empirical expressions to describe how *T* depends on *R*_s_ (which itself depends explicitly on material resistivity (*ρ*), *R*_in_, *R*_jxn_ and AR); approaches that typically require fitting parameters^35,37,38^, or using other empirically sourced quantities to achieve agreement between experimental and simulated data^13,20,39,40^. The *T*-*R*_s_ curve has a characteristic shape, which was highlighted by Mutiso *et al*.^38^ who fit an empirical expression with good agreement to a percolative model derived from thin-films^40^. However, none of these semi-empirical approaches are predictive, nor can they accurately describe a wide range of NWNs.

In this work we use the Mie light scattering theory (MLST) of NWNs described by Khanarian *et al*. to predict the transmittance as a function of the diameter of the NWs and the surface fraction coverage^41^. MLST is an exact theory which has no fitting parameters and is only dependent on the wavelength of incident light, the NW diameter, and the optical constants of the NW material. We build upon the electrical MNR model by incorporating a first principles approach based on MLST of NWs to determine the electro-optical performance of the NWN. This fully predictive model establishes the limits of Ag NWN performance and faithfully captures the behaviour of experimental data from the literature over a wide range of NW ARs. Importantly, this tool can be used to engineer NW systems for different applications in a true materials-by-design approach, allowing an effective comparison of different NWN processing methods that will facilitate the adoption of NWN films in current and next-generation devices.

## Results

**Figure 2:**
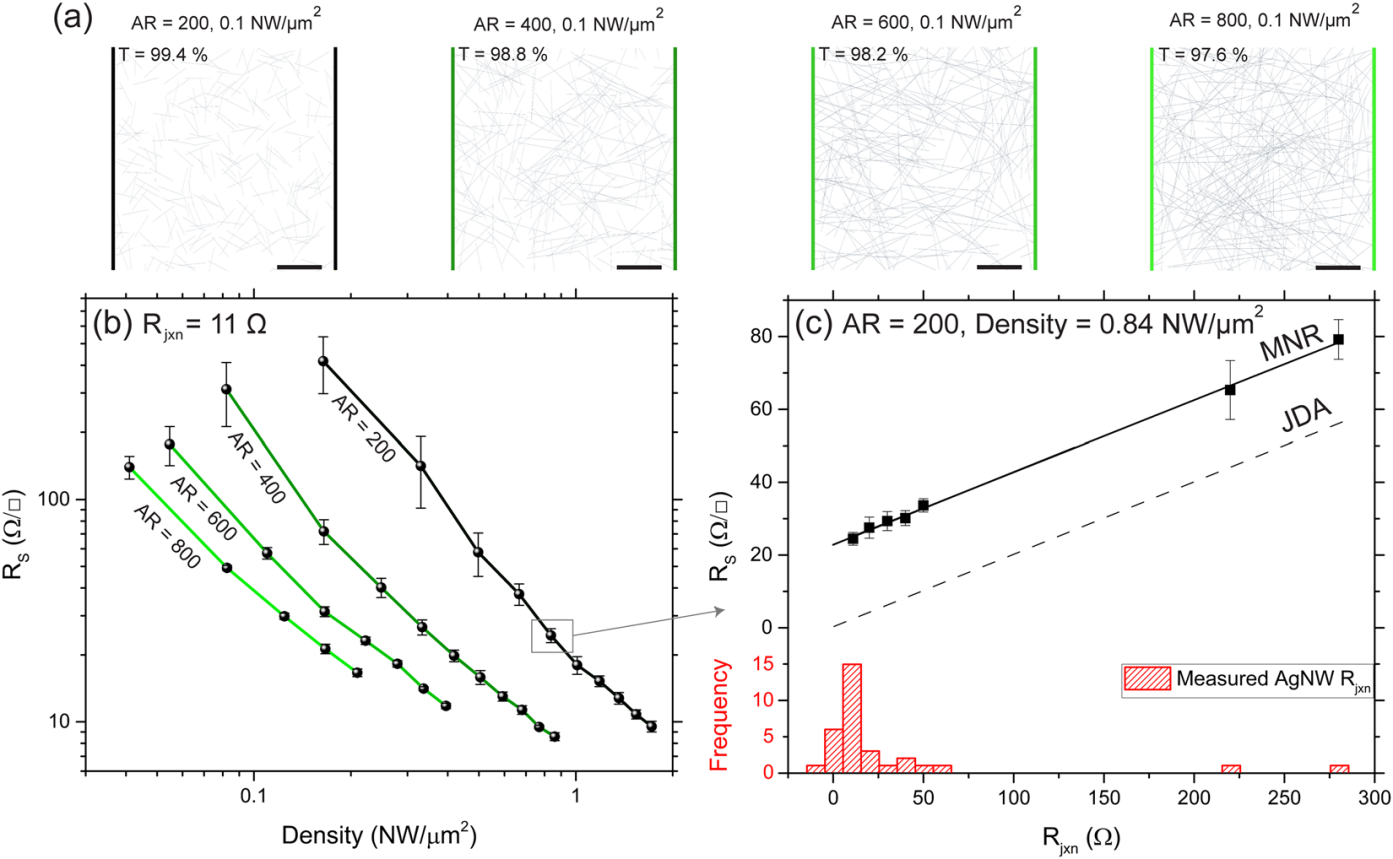
(**a**) Representative plots of simulated wire networks at a density of 0.1 NWs/*µ*m^2^ for aspect ratios (ARs) of 200, 400, 600 and 800 with nanowire (NW) diameter of 30 nm and a simulated cell size of 50 × 50 *µ*m; the respective transmittance (*T*) values are shown on each panel. (**b**) Average sheet resistance (*R*_s_) as a function of the Ag nanowire network density for four AR values where the NW-NW junction resistance (*R*_jxn_) is 11 Ω and the NW material  resistance is included using the multi-nodal representation (MNR) model. The Ag NW diameter was fixed at 30 nm for all simulations, the error bars arise from the standard deviation of 10 simulated networks, the simulated cell size for the above ARs was 15, 30, 45 and 55 µm, respectively. (**c**) For the highlighted density of 0.84 NW/*µ*m^2^ with AR = 200 in (**b**), the average *R*_s_ was calculated as a function of the *R*_jxn_ using the MNR model, and when the inner resistance is neglected from the calculations in the junction dominated approach (JDA). Below the main plot is a histogram of the experimentally measured *R*_jxn_ distribution of Ag NWs from Bellew *et al*.^42^.

The electrical performance of percolating NWNs depends significantly on the properties of the constituent NWs. Physical properties such as length and diameter determine the ultimate conductance potential of the network. The electrical performance of a Ag NWN was simulated using the MNR model for a given AR and wire density, setting *ρ* = 22.6 nΩm^23^, and *R*_jxn_ = 11 Ω corresponding to the median value of the experimentally optimised distribution of junction resistances in Ag/PVP systems (see Fig. 2c)^42^. For the purposes of simulation, the NWs were considered as rigid rods. Singular values of NW length and diameter were used in all computations, although the MNR model is also able to account for more realistic aspects of networks, e.g. dispersion in physical parameters such as length, diameter, *R*_jxn_ and the presence of “outlier” junctions. Here, our goal is to avoid unnecessary complexity and show the raw capabilities of the model in describing and predicting real world performance without any fitting parameters; the flexibility of the model enables the incorporation of dispersion and other disorder elements in a straightforward fashion.

Figure 2(a) shows plots of the simulated Ag NWNs at a density of 0.1 NW/*µ*m^2^ for ARs of 200, 400, 600 and 800; which corresponds to *T* values of 99.4%, 98.8%, 98.2% and 97.6%, respectively. The diameter of the NWs was fixed at 30 nm and the simulation box was set at 50 × 50 *µ*m in each case. In Fig. 2(b), the power law dependence of the *R*_s_ on NW density is clear for all AR values plotted, consistent with percolative and closed form models of electrical transport within NWNs^22,38,43^. The importance of including the material resistance in the calculation of the *R*_s_ is highlighted in Fig. 2(c), where *R*_s_ is calculated as a function of the *R*_jxn_ for a junction dominated approach (JDA, dashed black line) NWs have no internal resistance. The MNR model which accounts for the resistance of the wire segments between junctions is shown as a solid black line. When *R*_jxn_ → 0, the MNR model provides the ultimate limit of the network *R*_s_, which for an AR of 200 with a density of 0.84 NW/*µ*m^2^ (*T* = 95%) is ~20 Ω/□. The distribution on the lower half of the panel represents the range of experimentally measured *R*_jxn_ values for Ag NWs^42^. As the *R*_jxn_ increases, the *R*_s_ increases linearly, therefore NWNs must be subjected to processing steps after network formation to reduce the contact resistance between the NWs. These results highlight the importance of *R*_jxn_ optimisation in high performance NWNs, moreover, it shows the significant contribution of the *R*_in_ to the overall *R*_s_, and that the true upper bounds of electrical performance can only be determined when the inner wire resistance is considered.

To estimate the *T* of a NWN, we begin by considering the extinction coefficient at normal incidence, *C*_ext_, which represents the amount of light scattered and absorbed by a single NW from Mie theory^44^. The area fraction (AF) describes the projection of the NWs per unit area of the substrate which is defined as the density, *N*, per unit area multiplied by the length (*L*) and diameter (*D*) of the NWs.

2 $$AF=N\times L\times D$$

When the thickness of the network is comparable to the diameter of the NWs, the transmittance, *T*, as derived by Khanarian *et al*. can be expressed as^41^,

3 $$T={e}^{-AF\times {C}_{ext}}$$

**Figure 3:**
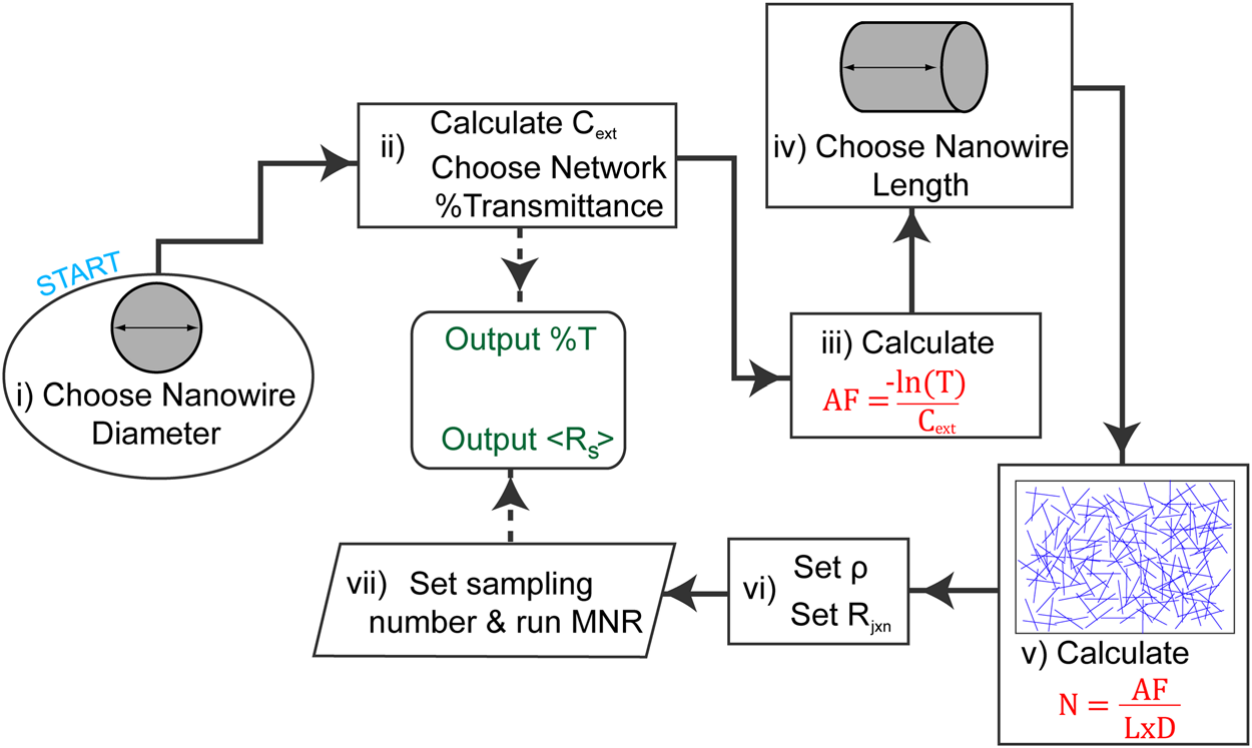
The flow diagram of the multi-nodal representation (MNR) and Mie light scattering theory (MLST) models for the electro-optical behaviours of nanowire networks (NWNs). (i) The diameter of the nanowire (NW) is selected; this is a critical parameter used in step (ii) to determine the extinction coefficient *C*_ext_. The desired optical transmittance (*T*) value of the NWN sample is also set here. (iii) Equation 3 is solved for the area fraction (AF) and (iv) the aspect ratio (AR) of the NWs is set by the NW length. (v) Equation 2 is solved for network density, *N*, which provides the NW density for the randomly generated network samples used in the MNR code. In (vi) the electrical properties, *ρ*, and the interwire junction resistance parameter (*R*_jxn_) is set. (vii) The MNR algorithm is executed and outputs an average sheet resistance <*R*_s_> for the prescribed number of network samples.

The flow diagram in Fig. 3 describes the implementation of the combined MNR and MLST models to simulate the electro-optical properties of NWNs. The only necessary inputs to the model are the NW diameter and length. The model then calculates the corresponding network density and determines the *R*_s_ given the values of *ρ* and *R*_jxn_. Thus the model not only predicts the performance of a particular network but given experimental *T*-*R*_s_ data for a network of known *D* and *L* values it can predict the average *R*_jxn_. The only way to alter the *R*_s_ for such a network (at a specific value of *T*) is to vary the *R*_jxn_ value used in the MNR model, which, as previously discussed, can be influenced by different processing techniques. A discussion and analysis of previously reported, empirically derived, electro-optical models is presented in the Supplementary Information as Figs S1 and S2. This data shows that the MNR MLST model describes the expected shape of not only experimentally obtained *T*-*R*_s_ data, but can accurately describe the synthetic data generated from previously reported semi-empirical models.

**Figure 4:**
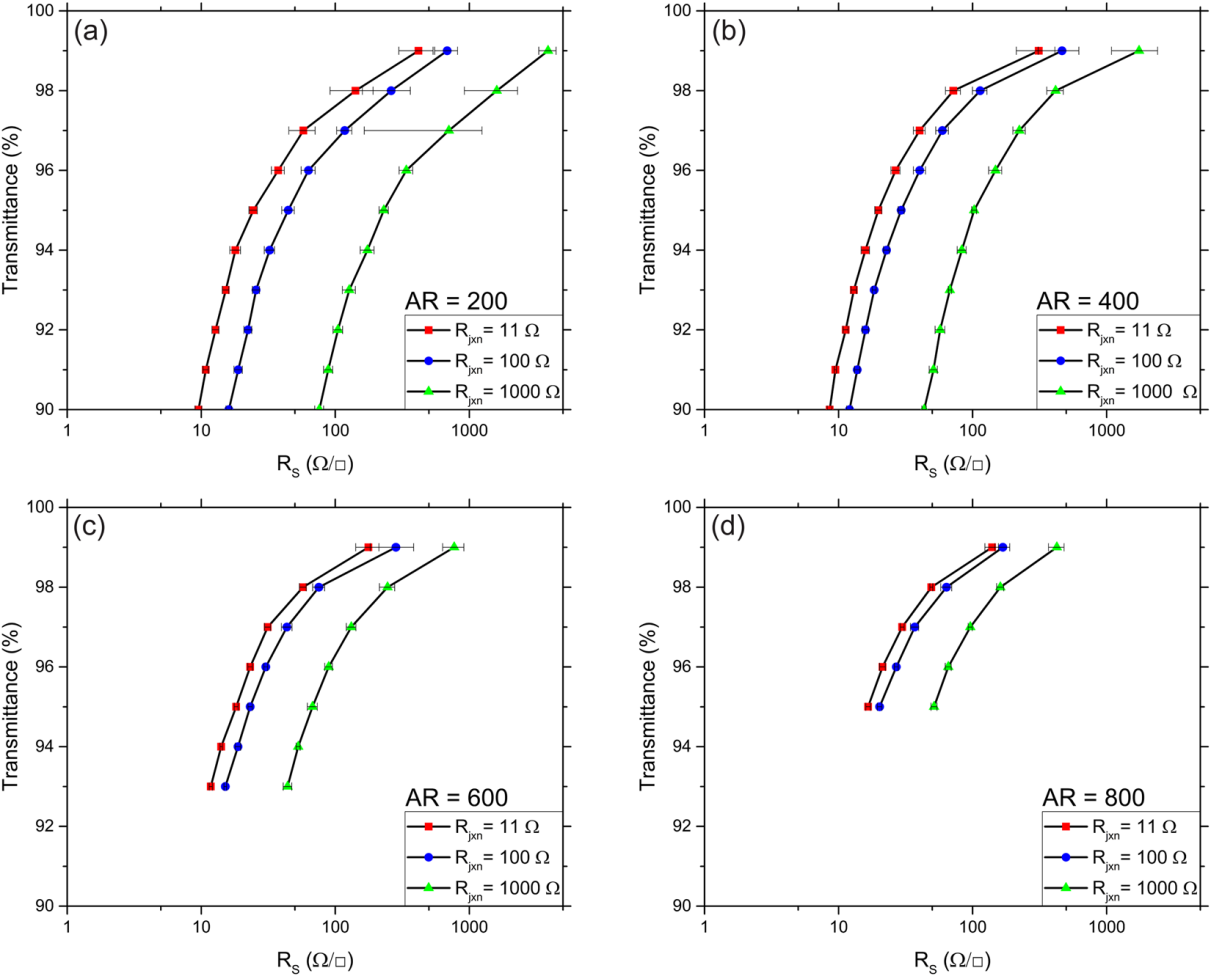
(**a–d**) Calculated sheet resistance (*R*_s_) for four aspect ratios (ARs) using the multi-nodal representation (MNR) and Mie light scattering theory (MLST) models for junction resistance (*R*_jxn_) values of 11, 100 and 1000 Ω. Panels (**c,d**) have less points due to machine memory limitations. The error bars arise from the standard deviation of 10 Ag nanowire networks (NWNs) for each data point. The transmittance is calculated using Eqs 2 and 3 of the Mie theory method which is explained in the main text.

Figure 4(a–d) shows the MNR calculated plots of the *T-R*_s_ for Ag NWNs with various AR values and for mean *R*_jxn_ values of 11 Ω, 100 Ω, 1000 Ω and in the case of perfect junctions with a resistance of 0 Ω. Each point in Fig. 4 was calculated by following the process outlined in Fig. 3 where *ρ* is fixed, and *R*_jxn_ is varied. Figure 4(a) highlights the importance of optimising *R*_jxn_ in the technologically relevant *T* region. It is important to note that when AR > 200 (Fig. 4(a,b)) at a *T* = 90%, the simulations predict that the *R*_s_ will be <100 Ω/□, which is acceptable for touch screen applications, however, solar cells and OLED electrodes require much lower *R*_s_ (~10 Ω/□) which can only be achieved by *R*_jxn_ optimisation (*R*_jxn_ < 100 Ω) of highly transparent networks (*T* > 95%). The truncation of the simulation in Fig. 4(c,d) is due to the prohibitive computational requirements to calculate sufficiently dense networks with *T* < 93% for AR = 600 and *T* < 95% for AR = 800 samples. The linear dependence of *T* with respect to the network density is plotted in Fig. S3, a dependence that is experimentally observed and has been theoretically derived by Ainsworth *et al*.^39^.

**Figure 5:**
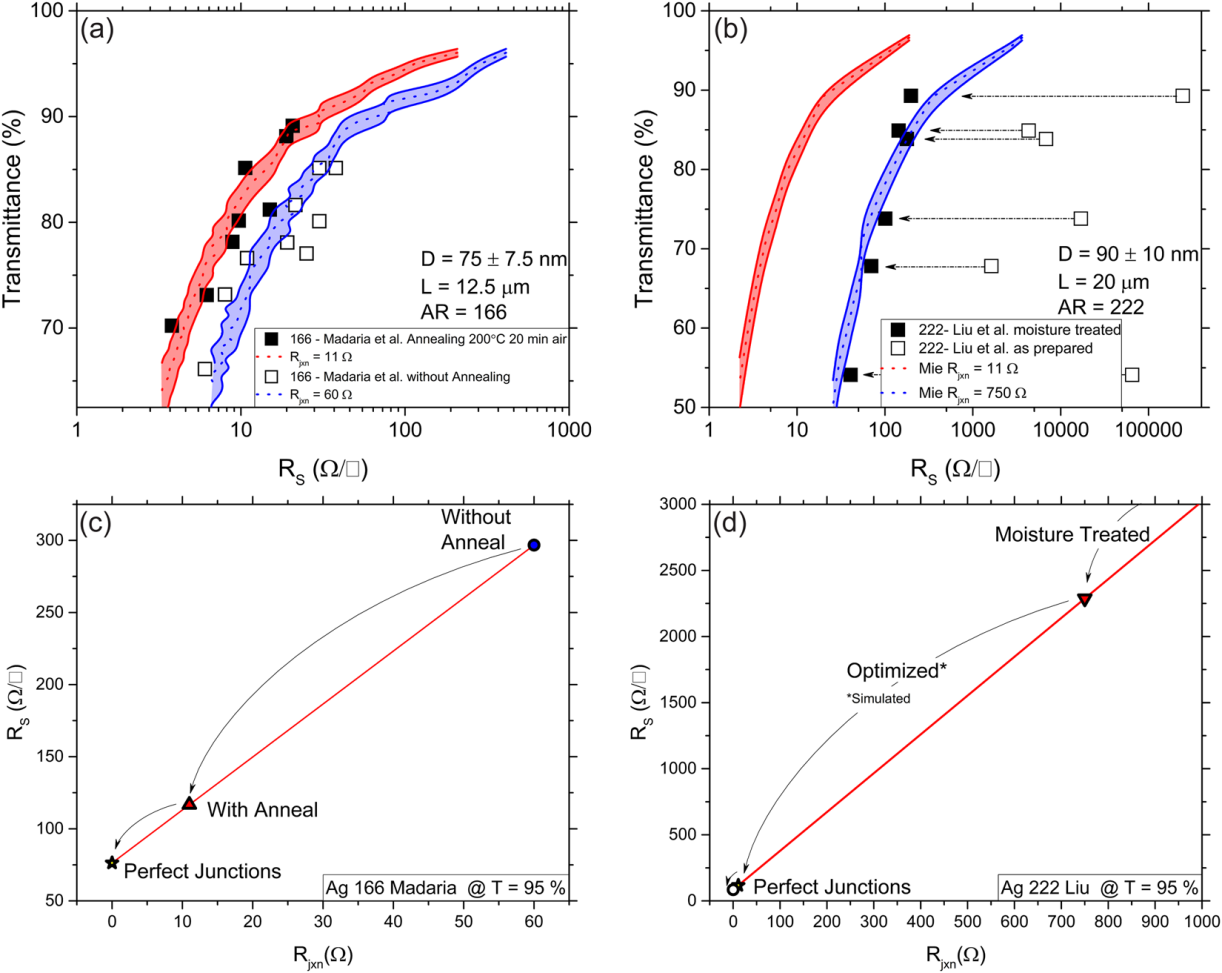
(**a,b**) The square data points on each panel represent experimental data of Ag nanowire networks (NWNs) taken from reported literature values with various aspect ratios (ARs) and post-deposition processing methods. The hollow data points in (**a**) show Ag NWNs which were measured after being deposited, while the black squares show data for NWN samples which were annealed for 20 minutes at 200 °C. In (**b**), the hollow squares show the electro-optical performance of as-prepared Ag NWNs with the black squares showing an increase in performance after the same NWNs were moisture treated. The dashed colored lines show the average sheet resistance (*R*_s_) predictions of the performance of the wire systems simulated using the multi-nodal representation (MNR) model where, in red, the junction resistance (*R*_jxn_) is set to 11 Ω and in blue the *R*_jxn_ was increased to (**a**) 60 Ω, and (**b**) 750 Ω to fit the experimentally reported data. The red and blue shaded areas bounded by the continuous line represents the standard deviation of the *R*_s_ of 10 simulated samples, and the error associated in the transmittance (*T)* calculation from Mie theory using the reported spread of diameter values. (**c,d**) *R*_s_ vs *R*_jxn_ analysis performed at *T* = 95% for the NWN in (**a,b**), respectively. In (**c**), a significant decrease in the *R*_s_ is observed when the NWNs are annealed, and in (**d**) the as-deposited network (data point not shown) is estimated to have *R*_s_ = 100 kΩ which yields by linear extrapolation *R*_jxn_ values of 34 kΩ. Moisture treated NWNs have a much lower *R*_s_, but simulations suggest this could be further optimised as *R*_jxn_ = 11 Ω. The ultimate limit of the *R*_s_ can be extrapolated by considering *R*_jxn_ → 0 where only the material resistance limits the electrical performance of the network.

In a physical NWN sample, it is impossible to probe individual *R*_jxn_ values via experimental means. A strength of the MNR model is that it provides insights into the average contribution of the *R*_jxn_ to the measured *R*_s_. By combining MNR and MLST models, we can begin to benchmark experimental data developing a materials-by-design approach to NWN-based TCs. Setting a theoretical benchmark for NWN systems allows a better comparison of synthesis methods, deposition procedures and specific post-processing techniques. For example, thermal annealing can hugely improve the *R*_s_ of as-deposited networks, but the anneal temperature and time must be chosen carefully and will depend on NW diameter and the thermal properties of the substrate. Figure 5(a) shows the improvement in the *R*_s_ by post-deposition annealing. Madaria *et al*.^45^ measured the performance of Ag NWs with AR = 166 pre and post anneal. The hollow square data points show the *R*_s_ before annealing the NWN films. The black squares show the marked decrease of the *R*_s_ after an anneal time of 20 min at 200 °C. The authors report the length and diameter values of the constituent NWs, which determine the densities of these networks (from Eq. 3) for the simulations. The MNR model can compute the electrical performance of these networks separating the *R*_s_ into the contributions of the wire material and the *R*_jxn_, the latter being varied to obtain a good fit to the experimental data. The dotted blue curve shows the average *R*_s_ results that match the as-deposited NWNs with *R*_jxn_ = 60 Ω. The width of the shaded coloured area represents both the horizontal component of the error from the standard deviation of the *R*_s_ for 10 simulated networks, and the vertical spread in *T* due to the uncertainty in the NW diameter value when calculating *C*_ext_. After the annealing step, the *T-R*_s_ curve is better described by *R*_jxn_ = 11 Ω, suggesting the annealing treatment has produced highly optimised TC films with extremely low *R*_s_ for that particular AR. However, even at this optimised *R*_jxn_ value, the NWNs fail the *R*_s_ requirement for photovoltaic applications and barely reaches the requirements for screen/lighting technologies (see Fig. 1).

Another example of NWN post processing benchmarking is shown in Fig. 5(b) using the data from Liu *et al*.^31^. In their study, moisture-induced capillary-forces were shown to cause a self-limiting cold welding of the NW junctions, hence reducing the *R*_s_. The hollow square data points show the *T*-*R*_s_ data of the as-prepared samples. The effect of the moisture treatment significantly reduces the *R*_s_ of the Ag NWNs and causes the network to adopt the “expected shape” curve which is an important indicator of the performance of the network as predicted by the MNR MLST models (cf. Fig. 4). We can apply the MNR MLST models to determine the *R*_jxn_ value needed to describe the network (comprised of NWs with AR = 222). The resulting blue shaded curve for the moisture treated samples has the expected *T-R*_s_ shape, and is well described by *R*_jxn_ = 750 Ω. The failure of the as-prepared film to exhibit the same shape as the moisture treated samples suggest that the network connectivity is poorly established or that there is a significant spread in *R*_jxn_ values. The power of MNR MLST is that it can determine the *R*_jxn_ values necessary to describe the measured *R*_s_ values in the as-prepared films - the spread is between 20 kΩ and 40 kΩ allowing a rapid evaluation of processing techniques used to form the network, which hitherto was not possible.

While it is obvious that moisture treatment has made a significant improvement to the measured *R*_s_, it has not produced the most optimised NWN yet. MNR MLST can predict the effect of additional optimisation. By decreasing the *R*_jxn_ to a value of 11 Ω (red shaded curve, Fig. 5(b)), the simulations suggest further room for improvement. *In-situ* resistance measurements during thermal annealing for NWNs of a similar diameter suggest that an annealing temperature of 200 °C is required to realise the most conducting NWN films^46^. Analysis of additional systems with AR = 182, 306, 440, 600, 641, 760, 800, 1000 and 2000 are shown in section 2 of the Supplementary Information confirming that our predictive model provides good agreement with experimental data. In some rare cases, MLST over-estimates the optical transmittance and, in some cases, MNR underestimates the *R*_s_. A discussion of deviations from the model are included in sections 3 and 4 of the Supplementary Information. Other factors that can affect the electro-optical predictions of our model include the inherent flexibility of the NWs and the diameter dependent persistence length which is known to play an important role in the connectivity of the networks^47,48^. At this point, for the sake of simplicity, our simulations only account for rigid rods and fixed persistence lengths. Nonetheless, the current model is flexible and can be extended to incorporate these elements.

Network optimisation can be graphically described by the combined MNR and MLST model. In Fig. 5(c,d), the *R*_s_ is calculated as a function of the *R*_jxn_ for the data presented in Fig. 5(a,b) at *T* = 95%. This linear relationship has been previously reported by our group when first implementing the MNR model and now serves as a roadmap for predicting the ultimate performance of NWN materials^23^. The linear decrease in the *R*_s_ assumes a decrease in the resistance of all junctions in the network, however in reality, some paths within the network may not be conducting initially and may require one of the processing steps previously discussed. As these additional paths become conducting, the simple linear relationship shown here may be curved or stepped^49,50^. The two datasets in Fig. 5(a,b) (two further examples for AR 306 and 760 are presented in Fig. S18) initially had *R*_jxn_ which were predicted to be higher than the optimised value. The ability of the model to separately consider junction and inner-wire resistances allows for the ultimate performance of the NWN to be determined, which occurs when *R*_jxn_ → 0 Ω. This allows an estimate of how close a NWN film is to having perfect interwire contacts, and enables a rigorous and quantitative analysis of post-processing techniques. While the tunability of the *R*_s_ in metallic NWNs via a combination of AR, NW density, material choice and *R*_jxn_ makes these materials attractive to numerous applications, the presence of a *R*_jxn_ between wires will always limit performance. Perfect lossless junctions may not be achievable in solution deposited NWNs but are a feature of seamless junction networks such as crackled template networks^51,52^.

## Conclusions

In this work, we combined two computational methods to deliver the first fully predictive model of both the electrical and optical performances of metal nanowire networks. The multi-nodal representation (MNR) model which calculates the sheet resistance (*R*_s_) of the nanowire networks (NWNs) considers both the resistance contribution of the nanowire segments and the nanowire (NW) junctions. Using experimentally measured resistivity and junction resistance values for the case of Ag NWNs, we show how the *R*_s_ depends on the nanowire length/diameter aspect ratio (AR). The inner-wire resistance is also important as it determines the lowest attainable *R*_s_, and the inclusion of the skeletal resistance allows the magnitude of the junction resistance between overlapping wires, *R*_jxn_, to reveal the level of optimisation of the NWN through post-processing steps. The Mie light scattering theory (MLST) model describes the optical transmittance of NWNs according to its fundamental physical properties and network density. This simple but robust model achieves excellent agreement with experimental data over a wide range of NW ARs. The results of this work show that simulation of NWNs is an important tool in benchmarking the efficacies of post-processing methods, and offers a strategic approach to exploring the potential applications of NWN materials and guiding the synthesis of systems for specific needs. NWNs are well suited as replacements for ITO (tin-doped indium oxide) in a wide variety of current and emerging flexible devices. The development of a predictive model for these materials is an important step towards a materials-by-design approach for transparent conductor applications.

## Methods

MNR simulations were implemented in the Python language, the code for which is available in the Supplementary Information. The networks were generated according to the input parameters of wire diameter, wire length, wire density (number of wires per unit area) and simulation box length, which defines the squared area of the box where wires are randomly placed. The simulation box was always set larger than two times the wire length. Ag NW material resistivity is *ρ* = 22.6 nΩm, and the *R*_jxn_ was varied according to the experiment. The MNR voltage grid scheme, which is described in detail in reference^23^ of the manuscript maps the spatial coordinates of the interwire connection points, and assigns either an interwire junction resistance *R*_jxn_, or an inner-wire segment with a resistance calculated by *R*_in_ = *ρl/A*, where *l* is the length of the segment and *A* is the cross-sectional area of the wire. The corresponding resistance matrix is solved using Kirchhoff’s circuit law to obtain the *R*_s_ of the sample. The number of representative NWN samples of the ensemble was set to 10. The program output the average *R*_s_, and the standard deviation of the *R*_s_.

The *C*_ext_ is calculated using the MatScat^53^ (Mie theory for infinite cylinders) implementation by Schäfer *et al*.^54^ and depends only on the NW diameter and the optical constants for the metal. The refractive index (*n*) and extinction coefficient (*k*) used for Ag is, *n* = 0.13936, *k* = 3.5604 at *λ* = 546 nm^55^. From Equations 2 and 3, the NWN density corresponds to a *T* value. *T*-*R*_s_ data of NWNs across a wide variety of aspect ratios was gathered from 17 publications, where the NW lengths and diameters were reported. The *T* values reported by these publications were converted into NWN densities which were calculated by MNR through the process outlined in Fig. 3. Where a spread in diameter values was reported, the upper and lower bounds described a variation in *C*_ext_ and hence *T* which is displayed as the shaded areas on the *T*-*R*_s_ graphs.

## Supplementary information

Supplementary information document

## Data Availability

All data generated or analysed during this study are included in this published article (and its Supplementary Information Files), the datasets are also available from the corresponding author on reasonable request.
