# Supplementary material for: The Electro-Optical Performance of Silver Nanowire Networks
Source: Sci Rep. 2019 Aug 9;9:11550. doi: 10.1038/s41598-019-47777-2 (PMC6689048; doi:10.1038/s41598-019-47777-2)
Supplement: Supplementary file 1 — Supplementary information document [file 41598_2019_47777_MOESM1_ESM.docx]

Supplementary Information

The Electro-Optical Performance of Silver Nanowire Networks

Hugh G. Manning, ^†, §, *^ Claudia Gomes da Rocha,^⊥^ Colin O’Callaghan,^φ, §^ Mauro S. Ferreira,^φ, §^ John J. Boland ^†, §^

† School of Chemistry, Trinity College Dublin, Dublin 2, Ireland

⊥ Department of Physics and Astronomy, University of Calgary,

2500 University Drive NW Calgary, Alberta T2N 1N4, Canada.

φ School of Physics, Trinity College Dublin, Dublin 2, Ireland.

§Centre for Research on Adaptive Nanostructures and Nanodevices (CRANN) & Advanced Materials and Bioengineering Research (AMBER) Research Centre,

Trinity College Dublin, Dublin 2, Ireland.

**Supplementary Figures**

1. **MNR Computational Code**

This code can be copied and pasted into Python, please note the required packages which are detailed below.

#!/usr/bin/env python

'''''

    File name: MNR_Model_NWN.py

    Authors: C. G. Rocha, C. O'Callaghan, H. G. Manning

    Date created: 01/10/2014

    Date last modified: 10/12/2018

    Python Version: 2.7

    This program is free software: you can redistribute it and/or modify

    it under the terms of the GNU General Public License as published by

    the Free Software Foundation, either version 3 of the License, or

    (at your option) any later version.

    This program is distributed in the hope that it will be useful,

    but WITHOUT ANY WARRANTY; without even the implied warranty of

    MERCHANTABILITY or FITNESS FOR A PARTICULAR PURPOSE. See the

    GNU General Public License for more details.

    You should have received a copy of the GNU General Public License

    along with this program. If not, see <https://www.gnu.org/licenses/>.

    Required packages: numpy, matplotlib, scipy, math, itertools, shapely, networkx, cvxopt, collections.

    Additionally, this code uses a sparse linear algebra package named symmlq.py developed by the Systems Optimisation Laboratory (SOL)

    at Stanford University, Department of Management Science and Engineering (MS&E), which is available at

    <http://stanford.edu/group/SOL/software.html> under the terms of The MIT License (MIT). In some cases, minres.py (also developed

    by the SOL) provides better results.

Citation:

    If you use this code in academic publications, please, cite our work appropriately.

'''

**from** shapely.geometry **import** LineString, MultiLineString, MultiPoint, Point

**from** shapely.ops **import** cascaded_union

**from** scipy.misc **import** comb

**from** itertools **import** product

**import** scipy.stats as stats

**import** matplotlib.pyplot as plt

**import** matplotlib.patches as patches

**import** math

**import** numpy as np

**from** itertools **import** islice

**from** cvxopt **import** matrix, solvers

**from** cvxopt.base **import** sparse

**from** cvxopt.base **import** matrix as m

**from** cvxopt.lapack **import** *

**from** cvxopt.blas **import** *

**import** cvxopt.misc as misc

**from** symmlq **import** *

**import** networkx as nx

**from** itertools **import** islice, combinations

**from** collections **import** Counter, defaultdict

**def** Gfun(x,y,trans='N'):

    '''''Function that passes matrix A to the symmlq routine which solves Ax=B.'''

    gemv(Amatrix,x,y,trans)

'''''

Parameters:

-----------

   R_junc: float

           Junction resistance value (in Ohms).

   rho0: float

         Wire resistivity (in Ohms-um).

  wire_diameter: float

                  Wire diameter (in nm)

   wire_length: float

                Wire length (in um).

   box_length: float

               Squared area of box_length x box_length where wires will be randomly spread (in um).

               Electrodes are placed on the left and right side of the box and have same dimensions as the size of the box (elec_length).

   samples: int

            Number of representative NWN samples of the ensemble.

   n_initial: float

              A scan in wire densities is performed. This is the initial wire density value (in #wires/um^2).

   n_final: float

            A scan in wire densities is performed. This is the final wire density value (in #wires/um^2).

   nstep: float

          A scan in wire densities is performed. This is the wire density step (in #wires/um^2).

   distl: boolean

         Stablishes if a dispersion in wire lengths is to be considered. For a network made of wires all with the same length wire_length,

         distl = False. Otherwise, distl = True which enables a truncated normal distribution of lengths with average length of lmean, standard deviation

         of sigmal, truncated at [lower_l,upper_l].

   A0: float

       Cross-sectional area of a cylinder (in um^2).

Returns: 'output.txt' (text file)

         four columns output file containing: wire density (density), average sheet resistance (resAvg), standard deviation in the sheet resistance (resStd), junction density (junc_avg).

'''

# Start ---------- Parameters block -------------

R_junc = 11.0

rho0 = 0.0226

wire_diameter = 30.0

wire_length= 6.0

box_length = 15.0

samples = 10

elec_length = box_length

box_y = box_length

lead_sep = box_length

n_initial = 0.16411

n_final = 1.90079

nstep = 0.16411

distl = False

lower_l = 2.2

upper_l = np.inf

sigmal = 2.0

lmean = wire_length

A0 = math.pi*((wire_diameter*0.001)/2)**2

# End ---------- Parameters block -------------

# ---------- Output file -------------

res_file = "output.txt"

res_dist = open(res_file,"w")

# ---------- Parameters for symmlq routine -------------

tol=1e-10

show=False

maxit=None

# ---------- Auxiliary lists for ensemble calculation -------------

res_list=[]

short_sep_list=[]

junc_dens=[]

dens_temp=[]

avg_res_temp=[]

st_dev_temp=[]

**for** density **in** np.arange(n_initial,n_final,nstep):

**for** sample **in** range(samples):

        area = box_length**2 # box area (in um^2)

        box_x = box_length # box width (in um)

        box_y = box_length # box length (in um)

        num_junc = 0 # junction counter

        nwires = area*density # total number of nanowires

        # Start ---------- Creation of random stick coordinates and electrodes -------------

                # a single wire is represented by a set of initial and final coordinates as [(x1,y1),(x2,y2)].

        x1 = np.random.rand(int(nwires))*box_x

        y1 = np.random.rand(int(nwires))*box_y

        length_array = np.zeros(int(nwires))

**if** distl == True:

            lengths = stats.truncnorm((lower_l - lmean) / sigmal, (upper_l - lmean) / sigmal, loc=lmean, scale=sigmal)

            length_array = lengths.rvs(size=nwires)

**else**:

            length_array.fill(wire_length)

        # Sorting the angles that define the wire orientation (in radians from 0 to 2 *pi).

        theta1 = np.random.rand(int(nwires))*2.0*math.pi

        x2 = length_array * np.cos(theta1) + x1

        y2 = length_array * np.sin(theta1) + y1

        # Adding to the coordinate list (x1,y1) the points corresponding to the contact leads.

        x1 = np.insert(x1, 0, 0.0)

        x1 = np.insert(x1, 0,0)

        # Adding to the coordinate list (x2,y2) the points corresponding to the contact leads.

        x2 = np.insert(x2, 0, 0.0)

        x2 = np.insert(x2, 0,0)

        ypostop = box_y/2 + elec_length/2

        yposbot = box_y/2 - elec_length/2

        y1 = np.insert(y1, 0,ypostop)

        y1 = np.insert(y1, 0,ypostop)

        y2 = np.insert(y2, 0,yposbot)

        y2 = np.insert(y2, 0, yposbot)

        xposleft = box_x/2-lead_sep/2

        xposright = box_x/2+lead_sep/2

        x1[0]= xposleft

        x2[0] = xposleft

        x1[1] = xposright

        x2[1] = xposright

        # Merging [(x1,y1),(x2,y2)] in accordance to shapely format.

        coords1 = zip(x1,y1)

        coords2 = zip(x2,y2)

        coords = zip(coords1,coords2)

        mlines = MultiLineString(coords)

        nwires_plus_leads = int(nwires+2)

                # End ---------- Creation of random stick coordinates and electrodes -------------

                # Start ---------- Identifying intersections between wires -------------

        # all pair wire combination

        lines_comb = combinations(mlines, 2)

        # list storing True or False for pair intersection

        intersection_check = [pair[0].intersects(pair[1]) **for** pair **in** lines_comb]

        # list storing the indexes of intersection_check where the intersection between two wires is TRUE

        intersections = [i **for** i, x **in** enumerate(intersection_check) **if** x]

        # full list containing all non-repeated combinations of wires

        combination_index = list((i,j) **for** ((i,_),(j,_)) **in** combinations(enumerate(mlines), 2))

        # list storing the connection (wire_i, wire_j)

        intersection_index = [combination_index[intersections[i]] **for** i **in** range(len(intersections))]

        # checking the coordinates for interesection points

        inter_point_coll = [pair[0].intersection(pair[1]) **for** pair **in** combinations(mlines, 2)]

        # eliminating empty shapely points from the previous list

        no_empty_inter_point_coll = [inter_point_coll[intersections[i]] **for** i **in** range(len(intersections))]

        # total number of intersections

        nintersections = len(intersection_index)

                # End ---------- Identifying intersections between wires -------------

                # Start ---------- MNR nodal mapping -------------

        # dictionary containing wire index: [list of wires connected to a given wire]

        wire_touch_list = defaultdict(list)

**for** k, v **in** intersection_index:

            wire_touch_list[k].append(v)

            wire_touch_list[v].append(k)

        # dictionary containing wire index: [label nodes following MNR mapping]

        wire_touch_label_list = defaultdict(list)

        each_wire_inter_point_storage = defaultdict(list)

        label = 2

        # Assigning new node labelling according to MNR mapping

**for** i **in** iter(wire_touch_list.viewitems()):

**for** j **in** range(len(i[1])):

                cpoint = mlines[i[0]].intersection(mlines[i[1][j]])

                npoint = (cpoint.x,cpoint.y)

                each_wire_inter_point_storage[i[0]].append(npoint)

**if** i[0] > 1:

                    wire_touch_label_list[i[0]].append(label)

                    label += 1

**else**:

                    wire_touch_label_list[i[0]].append(i[0])

                maxl = label # dimension of the resistance matrix

        # flattening intersection_index for counting the amount of occurances of wire i

        flat = list(sum(intersection_index, ()))

        conn_per_wire = Counter(flat)

        # checking for isolated wires

        complete_list = range(nwires_plus_leads)

        isolated_wires = [x **for** x **in** complete_list **if** **not** x **in** flat]

        # list containing the length segments of each wire (if it has a junction)

        each_wire_length_storage = [[] **for** _ **in** range(nwires_plus_leads)]

        # Routine that obtains the segment lengths on each wire

**for** i **in** each_wire_inter_point_storage:

            point_ini = Point(mlines[i].coords[0])

            point_fin = Point(mlines[i].coords[1])

            wlength = point_ini.distance(point_fin)

            wire_points = each_wire_inter_point_storage[i]

            dist = [0.0]*(len(wire_points)+1)

**for** j **in** range(len(wire_points)):

                point = Point(wire_points[j])

                dist[j] = point_ini.distance(point)

            dist[-1] = wlength

            dist.sort()

            dist_sep = [0.0]*len(dist)

            dist_sep[0] = dist[0]

            dist_sep[1:len(dist)] = [dist[k]-dist[k-1] **for** k **in** range(1,len(dist))]

            each_wire_length_storage[i].append(dist_sep)

                # End ---------- MNR nodal mapping -------------

                # The MNR mapping associated to the NWN is also converted into a mathematical graph given by G.

                # G contains 2*nintersections nodes and we conventioned that left and right electrodes are labelled as node 0 and 1, respectively.

        G = nx.Graph()

        G.add_nodes_from(range(2*nintersections))

        mr_matrix_plus = np.zeros((2*nintersections,2*nintersections))

        inner_count = 0

        inter_count = 0

                # Start ---------- Building resistance matrix -------------

**for** iwire **in** xrange(nwires_plus_leads):

**if** each_wire_inter_point_storage[iwire]:

**for** j, pointj **in** enumerate(each_wire_inter_point_storage[iwire]):

                    point = Point(pointj)

**for** i, pointw **in** enumerate(each_wire_inter_point_storage[iwire]):

                        comp_pointw = Point(pointw)

                        inter_dist = point.distance(comp_pointw)

                        round_inter_dist = round(inter_dist, 4)

**for** il **in** each_wire_length_storage[iwire][0]:

                            value = float(il)

                            value = round(value,4)

**if** value == round_inter_dist **and** value != 0:

                                inner_resis = (float(value) * rho0 / A0)

**if** iwire != 0 **and** iwire != 1 **and** mr_matrix_plus[wire_touch_label_list[iwire][i], wire_touch_label_list[iwire][j]] == 0.0:

                                    mr_matrix_plus[wire_touch_label_list[iwire][i], wire_touch_label_list[iwire][j]] = -1.0/inner_resis

                                    mr_matrix_plus[wire_touch_label_list[iwire][j], wire_touch_label_list[iwire][i]] = -1.0/inner_resis

                                    G.add_edge(wire_touch_label_list[iwire][i],wire_touch_label_list[iwire][j])

                                    inner_count += 1

**for** k, label **in** enumerate(wire_touch_list[iwire]):

**for** kk, pointk **in** enumerate(each_wire_inter_point_storage[label]):

                            pointk = Point(pointk)

                            inter_dist = point.distance(pointk)

                            round_inter_dist = round(inter_dist, 4)

**if** round_inter_dist == 0 **and** mr_matrix_plus[wire_touch_label_list[iwire][j], wire_touch_label_list[label][kk]] == 0:

                                G.add_edge(wire_touch_label_list[label][kk],wire_touch_label_list[iwire][j])

                                r0 = -1/R_junc

                                mr_matrix_plus[wire_touch_label_list[iwire][j], wire_touch_label_list[label][kk]] = r0

                                mr_matrix_plus[wire_touch_label_list[label][kk], wire_touch_label_list[iwire][j]] = r0

        sum_rows_mr_plus = mr_matrix_plus.sum(1)

        np.fill_diagonal(mr_matrix_plus, abs(sum_rows_mr_plus))

        mr_nozero_rows_plus = mr_matrix_plus[~(mr_matrix_plus==0).all(1),:]

                # nonconnected wires are eliminated from the resistance matrix

        mr_nonconnected_plus = mr_nozero_rows_plus[:,~(mr_nozero_rows_plus==0).all(0)]

        # End ---------- Building resistance matrix -------------

                # input current vector

        i0 = 1.0 # absolute value of the current (in Amp)

        ic = np.zeros(mr_nonconnected_plus.shape[0])

        ic[0] = +i0

        ic[1] = -i0

        Imatrix = m(ic)

                # Solving Ohm's law in matrix form, R^(-1)V = I. Resulting voltages are in Volts.

        Amatrix = m(mr_nonconnected_plus)

        elec_pot_mr = symmlq(Imatrix, Gfun, show=show, rtol=tol, maxit=maxit)

                # Sheet resistance

        resistance = ((elec_pot_mr[0][0] - elec_pot_mr[0][1]))/i0

                # Checking if there is a path connecting electrodes at nodes 0 and 1

**if** nx.has_path(G,0,1):

            separation_short = nx.shortest_path_length(G,0,1)

            res_list.append(resistance)

            short_sep_list.append(separation_short)

            junc_dens.append(float(nintersections)/area)

    junc_avg = np.mean(junc_dens)

    resAvg = np.mean(res_list)

    resStd = np.std(res_list)

    short = np.mean(short_sep_list)

    dens_temp.append(junc_avg)

    avg_res_temp.append(resAvg)

    st_dev_temp.append(resStd)

    res_dist.write("%s %s %s %s\n" %(density,resAvg,resStd,junc_avg))

**print** "Density: %s, Average resistance: %s, Standard deviation: %s, Junction density: %s" %(density,resAvg,resStd,junc_avg)

    res_list=[]

    short_sep_list=[]

    junc_dens=[]

res_dist.close()

**2. Calculation of optical transmittance**

The earliest electro-optical models related the transmittance, *T*, of NWNs to an appropriate film thickness which is akin to the method used for continuous metallic films but split into two distinct regimes; bulk like behaviour, and percolative behaviour. The equation which describes the latter,^1^

$T=\left[ 1+\frac{1}{\Pi}\left( \frac{Z_{0}}{R_{s}} \right)^{1/(n+1)} \right]^{-2}$ (s1)

uses Π as the percolative figure of merit, which is a dimensionless quantity relating the ratio of the DC conductivity of the film to the optical conductivity. *Z*_o_ is the impedance of free space (377 Ω), *R*_s_ is the measured sheet resistance, and *n* is the percolative exponent. Both Π and *n* are found by fitting Equation s1 with experimental data. This treatment of the network does not take into account the nanowire aspect ratio (AR) and has serious limitations of use. To correct this, the nanowire dimensions were more clearly linked through an area fraction (AF), which is the number density of the nanowires in the network area, multiplied by the projected area of the nanowire. The empirical relationship between the AF and the optical transmittance at λ = 500 nm is given by,^2^

$T=100-a_{1}AF$ (s2)

where a_1_ represents a fitting factor that accounts for all diameter and wavelength-dependent optical properties of the system and is also found through fitting the equation to experimental data.

The shape of the fits these models produce on a *T*-*R*_s_ curve are quite similar; this is highlighted by Mutiso *et al*.^3^ who fit synthetic data to both of these models with good agreement, and showed it can describe experimentally measured results of similar ARs. The circular data points in Figure S1 represent their computational data treated with Equation s2 for nanowire systems of different ARs. The dashed lines show a fit by the percolative model which can describe the shape of this data (and experimental data which is presented in their original manuscript).

Knowing just the dimensions of the nanowires used in this study for an AR of 50, 275 and 800, respectively, we can use MNR MLST and the process outlined in the main text to calculate the AF for the *T* values obtained by Mutiso *et al*.^3^ NWNs of this AF were generated and solved using the MNR model, varying the junction resistances (*R*_jxn­_) until a good agreement (± 10% for a 5-sample average at each data point) to the Mutiso *et al*. data was reached. The “parameter-space exploration” or optimising of *R*_jxn_ to reproduce the Mutiso *et al*. dataset is shown in Figure S1 as the solid black, green and blue triangles for the AR data for 800 (red), 275 (orange) and 50 (gold) circular data points. The distribution of *R*_jxn_ values required to match the *R*_s_ for these networks is shown in Figure S2. For AR of 50 and 800, the distribution is quite broad, for AR of 275 the distribution is narrow and shifted towards lower values. The values obtained for AR = 275 resulted in more reasonable *R*_jxn_ values, kΩ junction resistances are not typically found in post-deposition processed networks. AR = 275 was the only AR compared to experimentally measured data in the manuscript of Mutiso *et al*. and so may better represent Ag NWN systems in comparison to the other AR data.

**Figure S1**. Calculated transmittance, *T* versus simulated sheet resistance (*R*_s_) (coloured circles) from Mutiso *et al*.^3^ for Ag nanowire networks (NWNs) with aspect ratio (AR) = 50 (gold circles, diameter (*D)* = 40 nm), AR = 275 (orange circles, *D* = 75 nm), and AR = 800 (red circles, *D* = 40 nm). We used an effective junction resistance (*R*_jxn_) of 2 kΩ, and fitting parameters a_1_ of 87 and 82 for *D*_­_ = 40 and 75 nm, respectively. Dashed lines represent fits to the data using the percolative model by De *et al*.^1^ Triangular data points represent the multi-nodal representation (MNR) Mie light scattering theory (MLST) data which use the same *D* and length (*L)* as the Mutiso *et al*. data, but varies the *R*_jxn_ until the *R*_s_ agrees within ± 10%. Error bars correspond to an ensemble of 10 representative NWN samples generated in the simulations.

**Figure S2**. A distribution of junction resistance (*R*_jxn_) values required by the multi-nodal representation (MNR) Mie light scattering theory (MLST) models to agree within 10% of the data in Figure S1. The blue, green and black columns show the *R*_jxn_­ required to fit aspect ratios (AR) = 50, 275 and 800, respectively. The mean of the distribution is 2.2 kΩ, the effective junction resistance used by Mutiso *et al.*^3^ was 2 kΩ.

The mean *R*_jxn_ of the AR = 50, 275, 800 samples are 3838 Ω, 667 Ω, and 2517 Ω, respectively. The mean *R*_jxn_ of the whole distribution is 2.2 kΩ which is remarkably close to the 2 kΩ “effective contact resistance” used by Mutiso *et al*. to describe the effective junction resistance of Ag NWNs.

**Figure S3**. The linear relationship between the nanowire network (NWN) density and the transmittance of light at λ = 550 nm for four aspect ratio (AR) values 200, 400, 600 and 800 where the nanowire (NW) diameter (*D)* = 30 nm. The simulated cell size for the ARs was 15, 30, 45 and 55 µm, respectively. The NW-NW junction resistance (*R*_jxn_) was set to 11 Ω and the NW inner resistance is included using the multi-nodal representation (MNR) model.

**3. MLST With Good Agreement for T, AR = 182, 306, 440, 600, 641,760, 800, 1000 and 2000**

The MNR MLST predictive model shows good agreement with experimental data of AR = 182, 306, 440, 600, 641,760, 800, 1000 and 2000 where the Ag NWNs have been subjected to a coating or a substantial post deposition processing treatment such as annealing or optical welding (cf. Figure S1). As discussed in the main text, processing steps ensure that interwire connections are homogeneous, and the *T-R*_s_ curves have shapes which can be fit by semi-empirical models, such as those in Equations s1 and s2, or predictively calculated by MNR MLST.

Figure S4 shows the red dashed line representing an optimised *R*_jxn_ of 11 Ω for the networks simulated by MNR and treated with MLST creating analogous of the NWN samples of Bergin *et al*.^2^ The experimental data cannot be described by MNR and MLST with an *R*_jxn­_ value of 11 Ω, as the model significantly overestimates the *T* of the experimental samples. When a *R*_jxn_ value of 1 kΩ is used, shown by the blue dashed line, the results of the MNR and MLST models have a more agreeable trend, predicting the expected performance of relatively sparser samples. The red and blue shaded areas, bounded by the solid red and blue lines represent the standard deviation of the *R*_s_ taken for 10 simulated samples, and the error associated in the transmittance calculation from Mie theory using the reported spread of diameter values. The increased *R*_jxn_ value is justifiable as these samples were not subjected to annealing and can be described by the *Mutiso et al*. model where the effective contact resistance was set to 2 kΩ. Moreover, the predictions of MNR and MLST are in line with other NWNs of similar ARs, for example, Figure 5 in the main text shows AR = 166 and AR = 222 with comparable performances.

Figure S4. The square data points represent experimentally measured results for Ag nanowire networks (NWNs) of aspect ratio (AR) = 182 reported by Bergin *et al*.^2^  The blue dashed line is the figure of merit determined by the multi-nodal representation (MNR) Mie light scattering theory (MLST) models at a junction resistance (*R*_jxn_) = 1 kΩ, whereas the red dashed line predicts an improved performance if *R*_jxn_­ is optimised to 11 Ω. The red and blue shaded areas bounded by the continuous lines represents the standard deviation of the sheet resistance (*R*_s_) taken from 10 simulated NWN samples, and the error associated in the transmittance calculation from Mie theory using the reported spread of diameter values.

**Figure S5**. The square data points represent experimentally measured results for Ag nanowire networks (NWNs) of aspect ratio (AR) = 306 reported by Large *et al*.^4^ These networks were spray coated onto glass microscope slides. The samples were not subjected to any post-processing steps. The blue dotted line shows the figure of merit determined by the multi-nodal representation (MNR) Mie light scattering theory (MLST) model with a junction resistance (*R*_jxn_) = 80 Ω, the red dotted line describes the sheet resistance (*R*_s_) values for a more optimised *R*_jxn_ = 11 Ω. The red and blue shaded areas bounded by the continuous lines represents the standard deviation of the *R*_s_ taken from 10 simulated NWN samples, and the error associated in the transmittance calculation from Mie theory using the reported spread of diameter values.

**Figure S6**. The square data points represent experimentally measured results for Ag nanowire networks (NWNs) of aspect ratio (AR) = 440 reported by Song *et al*.^5^ These networks were deposited by the spin coating method and then coated with a sol-gel TiO_2_ layer to improve thermal and chemical stability of the Ag NWN. The sheet resistance (*R*_s_) for these samples were taken after a five minute 80 °C anneal. The blue dotted line shows the figure of merit determined by the multi-nodal representation (MNR) Mie light scattering theory (MLST) models with a junction resistance (*R*_jxn_) = 75 Ω, and the red dotted line describes a more optimised *R*_jxn_ = 11 Ω. The red and blue shaded areas bounded by the continuous lines represents the standard deviation of *R*_s_ taken for 10 simulated NWN samples, and the error associated in the transmittance calculation from Mie theory using the reported spread of diameter values.

**Figure S7.** The square data points represent experimentally measured results for Ag nanowire networks (NWNs) of aspect ratio (AR) = 600 reported by Lee *et al*.^6^ The NWN was formed by vacuum filtration and then transferred to the target substrate. Ag NWNs were then subjected to a laser welding process to reduce nanowire-nanowire contact resistance. The red dotted line is the figure of merit determined by the multi-nodal representation (MNR) Mie light scattering theory (MLST) models with a junction resistance (*R*_jxn_) = 11 Ω. The red shaded area bounded by the continuous lines represents the standard deviation of the sheet resistance (*R*_s_) taken for 10 simulated NWN samples, and the error associated in the transmittance calculation from Mie theory using the reported spread of diameter values.

**Figure S8.** The square data points represent experimentally measured results for Ag nanowire networks (NWNs) of aspect ratio (AR) = 641 reported by Zhu *et al*.^7^ In the fabrication of these Ag NWNs, a glass substrate was placed on a hot plate at 92 °C and Ag nanowire (NW) ink was spread by Meyer rod printing. The film was then plasma treated for 1 hour with a power of 75 W to remove the PVP and weld the Ag NWs together. The red dotted line is the figure of merit determined by the multi-nodal representation (MNR) Mie light scattering theory (MLST) model with a junction resistance (*R*_jxn_) = 11 Ω. The red shaded area bounded by the continuous lines represents the standard deviation of the sheet resistance (*R*_s_) taken for 10 simulated NWN samples, and the error associated in the transmittance calculation from Mie theory using the reported spread of diameter values.

**Figure S9.** The square data points represent experimentally measured results for Ag nanowire networks (NWNs) of aspect ratio (AR) = 760 reported by Ackermann *et al.*^8^ In the fabrication of these Ag NWNs, a glass substrate dip coated from an ethanolic Ag nanowire (NW) dispersion. The film was then annealed at 120 °C in an oven for 10 minutes. The blue dotted line shows the figure of merit determined by the multi-nodal representation (MNR) Mie light scattering theory (MLST) models with a junction resistance (*R*_jxn_) = 400 Ω, and the red dotted line was taken for a more optimised *R*_jxn_ = 11 Ω. The red shaded area bounded by the continuous lines represents the standard deviation of the sheet resistance (*R*_s_) taken for 10 simulated NWN samples, and the error associated in the transmittance calculation from Mie theory using the reported spread of diameter values.

**Figure S10**. The square data points represent experimentally measured results for Ag nanowire networks (NWNs) of aspect ratio (AR) = 800 reported by Lan *et al*.^9^ Ag nanowire (NW) films were spin-coated onto polyethylene terephthalate (PET) substrates and dried at 120 °C for 5 minutes. The dried films were pressed at 25 MPa for 30 seconds and coated in a PVA solution to create a Ag NW/PVA film. The blue dotted line shows the figure of merit determined by the multi-nodal representation (MNR) Mie light scattering theory (MLST) models with a junction resistance *R*_jxn_ = 250 Ω, and the red dotted line was taken for a more optimised *R*_jxn_ = 11 Ω. The red and blue shaded areas bounded by the continuous lines represents the standard deviation of the sheet resistance (*R*_s_) taken for 10 simulated NWN samples, and the error associated in the transmittance calculation from Mie theory using the reported spread of diameter values.

**Figure S11**. The square data points represent experimentally measured results for Ag nanowire networks (NWNs) of aspect ratio (AR) = 1000 reported by Kim *et al*.^10^ Ag nanowire (NW) inks were prepared with exfoliated clay platelets (ratio 2:1 by weight) to improve the solution and coating properties of the inks. Ag NW inks were coated onto polyethylene terephthalate (PET) substrates using a wire-wound rod and dried at 100 °C for 2 minutes. The blue dotted line shows the figure of merit determined by the multi-nodal representation (MNR) Mie light scattering theory (MLST) models with a junction resistance (*R*_jxn_) = 300 Ω, and the red dotted line taken for a more optimised *R*_jxn_ = 11 Ω. The red and blue shaded areas bounded by the continuous lines represents the standard deviation of the sheet resistance (*R*_s_) taken for 10 simulated NWN samples, and the error associated in the transmittance calculation from Mie theory using the reported spread of diameter values.

**Figure S12**. The square data points represent experimentally measured results for Ag nanowire networks (NWNs) of aspect ratio (AR) = 2000 reported by Li *et al.*^11^ Ag nanowire (NW) inks were formulated with ethyl cellulose, ethyl alcohol, isopropyl alcohol, ethyl acetate, pentyl acetate, and toluene and coated onto substrates using a Meyer rod. The blue dotted line shows the figure of merit determined by the multi-nodal representation (MNR) Mie light scattering theory (MLST) models with a junction resistance (*R*_jxn_) = 1056 Ω, and the red dotted line was taken for a more optimised *R*_jxn_ = 11 Ω. The red and blue shaded areas bounded by the continuous lines represents the standard deviation of the sheet resistance (*R*_s_) taken for 10 simulated NWN samples, and the error associated in the transmittance (*T*) calculation from Mie theory using the reported spread of diameter values. The extreme aspect ratios of this system prohibited the simulation of dense samples below *T* = 98.8%.

**4. MLST overestimating the optical transmittance (AR = 175, 250, 1000)**

Overestimation of the optical transmittance by the MLST model is discussed in this section. The overestimation of the measured *T* by the first-principles model could be due to a number of unreported factors which require systematic investigation which was beyond the scope of this study. The overestimation of MLST suggests that these networks may have large distributions of *L*_­_ and *D*, the NW solution may have included Ag nanoparticles, which are commonly present in Ag NWN solutions, or some other source of heterogeneity capable of reducing the *T* of the sample. In these cases, the MLST overestimates the experimental transmittance by 10-15%, which cannot be reached by increasing the *R*_jxn_, i.e. shifting the simulation curves further to the right. These results are presented in the following Figures S13, S14, and S15.

**Figure S13.** The square data points represent experimentally measured results for Ag nanowire networks (NWNs) of aspect ratio (AR) = 175 reported by Cann *et al*.^12^ The red line was taken with the multi-nodal representation (MNR) Mie light scattering theory (MLST) models setting a junction resistance (*R*_jxn_) = 11 Ω. The red error bars represent the standard deviation in sheet resistance (*R*_s_) taken for 10 simulated NWN samples.  No error is present in the transmittance (*T*) of the simulated samples as the authors did not report the spread in diameter (*D)* and length (*L)* values of the constituent nanowires.

**Figure S14.** The square data points represent experimentally measured results for Ag nanowire networks (NWNs) of aspect ratio (AR) = 250 reported by Hu *et al*.^13^ The red line was taken using the multi-nodal representation (MNR) Mie light scattering theory (MLST) models setting a junction resistance (*R*_jxn_) = 11 Ω. The red shaded areas bounded by the continuous lines represents the standard deviation of the sheet resistance (*R*_s_) taken from 10 simulated NWN samples, and the error associated in the transmittance calculation from Mie theory using the reported spread of diameter (*D*) values. The dispersion in the nanowire length (*L*) values was not reported.

**Figure S15**. The square data points represent experimentally measured results for Ag nanowire networks (NWNs) of aspect ratio (AR) = 1000 reported by Park *et al*.^14^ The red line was taken using multi-nodal representation (MNR) Mie light scattering theory (MLST) models, setting the junction resistance (*R*_jxn_) = 11 Ω. The red error bars represent the standard deviation of the sheet resistance (*R*_s_) taken from 10 simulated NWN samples. No error is present in the transmittance (*T)* of the simulated samples as the authors did not report the spread in diameter (*D)* and length (*L)* values of the constituent nanowires.

**5. MNR underestimating T and sheet resistance (AR=153, 360)**

The underestimation of the *R*_s_ by MLST occurs in two cases; in the case of AR 153 by Mayousse *et al*.^15^ the discrepancy between MLST and experimental data is only a few %*T*. For the anomalously high-performing networks of Bellet *et al*.,^16^ the difference between MNR and the experimental data is ~ 5-10%, even when *R*_jxn_ is set to 1 Ω. These NWNs at AR = 360 outperform networks of AR = 1000 (as shown in Figure 1 of the main text).

**Figure S16**. The square data points represent experimentally measured results for Ag nanowire networks (NWNs) of aspect ratio (AR) = 153 reported by Mayousse *et al*.^15^ The red dashed line was taken using multi-nodal representation (MNR) Mie light scattering theory (MLST) models, setting the junction resistance (*R*_jxn_) = 11 Ω, and the blue dashed line was taken for a more optimised value of just 1 Ω. The red and blue shaded areas bounded by the continuous lines represents the standard deviation of the sheet resistance (*R*_s_) taken from 10 simulated NWN samples, and the error associated in the transmittance (*T*) calculation from Mie theory using the reported spread of diameter values. The difference between the MLST and MNR model and the experimental data is quite small, but still the MLST model slightly underestimates the *T* performance of the experimental samples.

**Figure S17.** The square data points represent experimentally measured results for Ag nanowire networks (NWNs) of aspect ratio (AR) = 360 reported by Bellet *et al*.^16^ The red dashed line was taken using the multi-nodal representation (MNR) Mie light scattering theory (MLST) models setting the junction resistance (*R*_jxn_) = 11 Ω, and the blue dashed line was obtained for a more optimised value of just 1 Ω. The red and blue shaded areas bounded by the continuous lines represents the standard deviation of the sheet resistance (*R*_s_) taken from 10 simulated NWN samples, and the error associated in the transmittance calculation from Mie theory using the reported spread of diameter values.


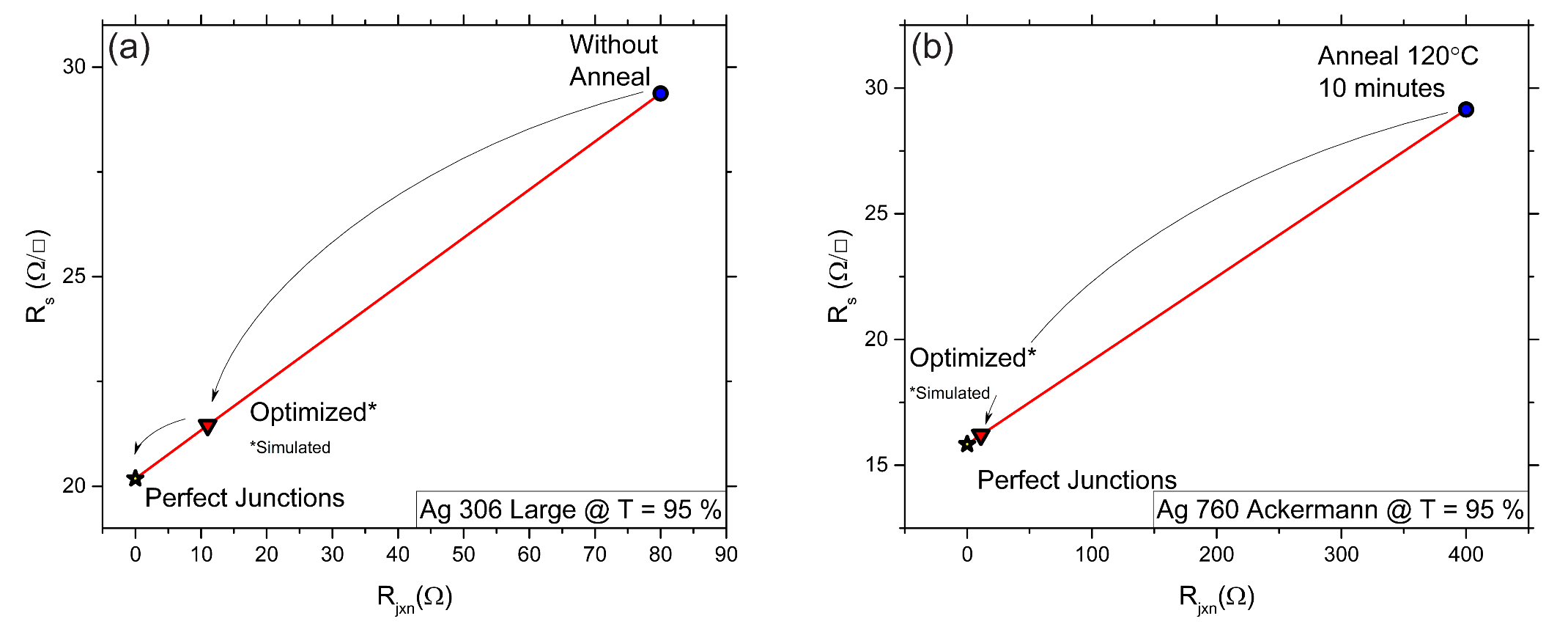


**Figure S18.** The ultimate sheet resistance of the reported datasets presented in Figures S1 and S5 at a transmittance (*T)* = 95%. In each case, the circular blue datapoint represents the junction resistance (*R*_jxn_) value required by the multi-nodal representation (MNR) Mie light scattering theory (MLST) simulations to match with the experimental data of (a) as-deposited Ag nanowire networks (NWNs) and (b) NWNs annealed at 120 °C for 10 minutes. The triangular red data points show the reported decrease of the theoretical sheet resistance (*R*_s_) of the Ag NWNs with optimised *R*_jxn_ values of 11 Ω. The ultimate sheet resistance can be extrapolated by considering *R*_jxn_ → 0 (limit of perfect junctions) in which only the material resistance limits the electrical performance of the networks, this is shown by the star shaped datapoint.

**References**

1 De, S., King, P. J., Lyons, P. E., Khan, U. & Coleman, J. N. Size effects and the problem with percolation in nanostructured transparent conductors. *ACS Nano* **4**, 7064-7072, doi:10.1021/nn1025803 (2010).

2 Bergin, S. M. *et al.* The effect of nanowire length and diameter on the properties of transparent, conducting nanowire films. *Nanoscale* **4**, 1996-2004, doi:10.1039/c2nr30126a (2012).

3 Mutiso, R. M., Sherrott, M. C., Rathmell, A. R., Wiley, B. J. & Winey, K. I. Integrating simulations and experiments to predict sheet resistance and optical transmittance in nanowire films for transparent conductors. *ACS Nano* **7**, 7654-7663, doi:10.1021/nn403324t (2013).

4 Large, M. J. *et al.* Predicting the optoelectronic properties of nanowire films based on control of length polydispersity. *Sci Rep-Uk* **6**, 25365, doi:10.1038/srep25365 (2016).

5 Song, T. B. *et al.* Highly Robust Silver Nanowire Network for Transparent Electrode. *ACS Appl Mater Interfaces* **7**, 24601-24607, doi:10.1021/acsami.5b06540 (2015).

6 Lee, J. *et al.* Very long Ag nanowire synthesis and its application in a highly transparent, conductive and flexible metal electrode touch panel. *Nanoscale* **4**, 6408-6414, doi:10.1039/c2nr31254a (2012).

7 Zhu, S. *et al.* Transferable self-welding silver nanowire network as high performance transparent flexible electrode. *Nanotechnology* **24**, 335202, doi:10.1088/0957-4484/24/33/335202 (2013).

8 Ackermann, T., Neuhaus, R. & Roth, S. The effect of rod orientation on electrical anisotropy in silver nanowire networks for ultra-transparent electrodes. *Sci Rep-Uk* **6**, 34289, doi:10.1038/srep34289 (2016).

9 Lan, W. *et al.* Ultraflexible Transparent Film Heater Made of Ag Nanowire/PVA Composite for Rapid-Response Thermotherapy Pads. *ACS Appl Mater Interfaces* **9**, 6644-6651, doi:10.1021/acsami.6b16853 (2017).

10 Kim, T. *et al.* Uniformly interconnected silver‐nanowire networks for transparent film heaters. *Advanced Functional Materials* **23**, 1250-1255, doi:10.1002/adfm.201202013 (2013).

11 Li, B., Ye, S., Stewart, I. E., Alvarez, S. & Wiley, B. J. Synthesis and Purification of Silver Nanowires To Make Conducting Films with a Transmittance of 99%. *Nano Lett* **15**, 6722-6726, doi:10.1021/acs.nanolett.5b02582 (2015).

12 Cann, M. *et al.* High performance transparent multi-touch sensors based on silver nanowires. *Materials Today Communications* **7**, 42-50, doi:10.1016/j.mtcomm.2016.03.005 (2016).

13 Hu, L., Kim, H. S., Lee, J. Y., Peumans, P. & Cui, Y. Scalable coating and properties of transparent, flexible, silver nanowire electrodes. *ACS Nano* **4**, 2955-2963, doi:10.1021/nn1005232 (2010).

14 Park, H. G. *et al.* Silver Nanowire Networks as Transparent Conducting Films for Liquid Crystal Displays. *Ecs Solid State Letters* **4**, R50-R52, doi:10.1149/2.0031510ssl (2015).

15 Mayousse, C., Celle, C., Fraczkiewicz, A. & Simonato, J. P. Stability of silver nanowire based electrodes under environmental and electrical stresses. *Nanoscale* **7**, 2107-2115, doi:10.1039/c4nr06783e (2015).

16 Bellet, D. *et al.* Transparent Electrodes Based on Silver Nanowire Networks: From Physical Considerations towards Device Integration. *Materials (Basel)* **10**, 570, doi:10.3390/ma10060570 (2017).
